# Intermittent symptomatic functional mitral regurgitation illustrated by two cases

**DOI:** 10.5830/CVJA-2015-026

**Published:** 2015

**Authors:** Alper Aydin, Tayfun Gurol, Ozer Soylu, Bahadir Dagdeviren

**Affiliations:** Department of Cardiology, Faculty of Medicine, Bahcesehir University, Istanbul, Turkey; Department of Cardiology, Faculty of Medicine, Bahcesehir University, Istanbul, Turkey; Department of Cardiology, Faculty of Medicine, Bahcesehir University, Istanbul, Turkey; Department of Cardiology, Faculty of Medicine, Bahcesehir University, Istanbul, Turkey

**Keywords:** mitral regurgitation, electromechanical delay, Doppler echocardiography, left bundle branch block, mitral insufficiency

## Abstract

Functional mitral regurgitation may have different haemodynamic consequences, clinical implications and treatment options, such as surgical or percutaneous interventions or implanting a pacemaker. Here we present two cases with haemodynamically significant intermittent functional mitral regurgitation as the underlying mechanism of heart failure. The cases underline the importance of a high index of suspicion in patients with intermittent heart failure, and a careful analysis of echocardiographic images with simultaneous ECG, in order to delineate systolic and diastolic mitral regurgitation.

## Abstract

Heart failure (HF) in patients with normal left ventricular ejection fraction accounts for half of the diagnoses of HF. Careful echocardiographic analysis with simultaneous ECG in two patients developing acute heart failure allowed identification of an unusual cause of HF with normal left ventricular ejection fraction (LVEF), but related to sudden reversible functional mitral regurgitation in the absence of significant coronary artery stenosis.

## Case 1

A 54-year-old female was admitted to hospital with acute pulmonary oedema. Her ECG showed sinus tachycardia with left bundle branch block (LBBB) morphology, with a rate of 125 beats per min (bpm). Her symptoms improved following spontaneous conversion to sinus rhythm without LBBB.

Two-dimensional echocardiography revealed concentric left ventricular (LV) hypertrophy with normal systolic function (LVEF 70%), with mild rheumatic mitral regurgitation (MR), mild left atrial dilatation (4.3 cm) and elevated pulmonary artery systolic pressure (50 mmHg). The tenting area of the mitral leaflets and the tenting length was measured as 3.9 cm^2^ and 1.3 cm, respectively. The mitral annular dimension was 4.2 cm.

The results of her laboratory examination were normal. Her medical history was unremarkable for cardiovascular disease and she was not taking any anti-arrhythmia drugs. Since her symptoms occurred again the following day, the echocardiographic examination was repeated. In the second study, the rhythm was sinus tachycardia with LBBB morphology. The QRS duration was 150 ms.

**Figure 1.**
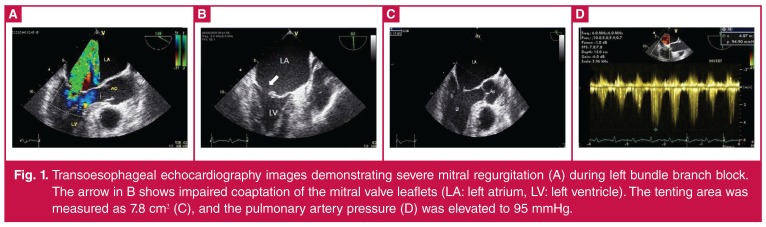
Transoesophageal echocardiography images demonstrating severe mitral regurgitation (A) during left bundle branch block. The arrow in B shows impaired coaptation of the mitral valve leaflets (LA: left atrium, LV: left ventricle). The tenting area was measured as 7.8 cm2 (C), and the pulmonary artery pressure (D) was elevated to 95 mmHg.

Transoesophageal echocardiography (TEE) revealed marked asynchronous contraction and dilatation of the left ventricle and atrium (5.1 cm). The left atrium was seen as being larger in this second assessment (5.1 cm), with severe MR (Fig. 1). The effective regurgitant orifice area was 0.6 cm^2^ with a regurgitant volume of 67 ml. The tenting area of the mitral leaflets and the tenting length were 7.8 cm^2^ and 1.8 cm, respectively. The mitral annular dimension was 4.8 cm.

**Figure 2.**
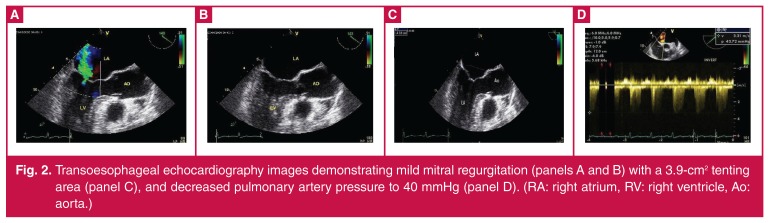
Transoesophageal echocardiography images demonstrating mild mitral regurgitation (panels A and B) with a 3.9-cm2 tenting area (panel C), and decreased pulmonary artery pressure to 40 mmHg (panel D). (RA: right atrium, RV: right ventricle, Ao: aorta.)

After spontaneous narrowing of the QRS duration to 60 ms, the third echocardiography showed mild MR with improved pulmonary pressure (Fig. 2). Ischaemic heart disease was excluded by coronary angiography. During follow up, paroxysmal LBBB recurred repeatedly, all with pulmonary oedema symptoms resolving with conversion to sinus rhythm without LBBB.

Because of the severe symptoms, which were resistant to medical therapy, we decided to perform mitral valve repair surgery. Mitral valvuloplasty with a rigid annuloplasty ring (Sorin Memo 3D 28-mm semi-rigid mitral) was performed. Within two years following the procedure, the patient had no symptoms of heart failure despite having paroxysmal LBBB attacks.

## Case 2

A 56-year-old female had complained of exertional dyspnoea and gradual intolerance during exercise for the previous three months. Transthoracic echocardiography (TTE), which was performed in another hospital, had revealed moderate-to-severe mitral regurgitation and therefore she was referred to have surgery for mitral valve replacement.

Her medical history was unremarkable for cardiovascular disease and she was not taking any anti-arrhythmia drugs. Initially, ECG revealed sinus rhythm with a heart rate of 66 bpm.

**Figure 3.**
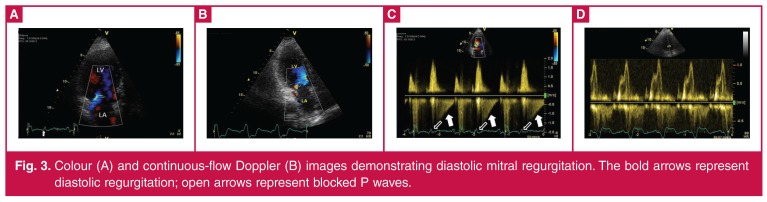
Colour (A) and continuous-flow Doppler (B) images demonstrating diastolic mitral regurgitation. The bold arrows represent diastolic regurgitation; open arrows represent blocked P waves.

TTE showed normal chamber volumes and functions with mild MR. During the TTE assessment she developed sudden dyspnoea with 2:1 atrio-ventricular (AV) block, and colour Doppler echocardiography revealed moderate early and mid-diastolic mitral regurgitation jets (V_max_ = 1.2 m/s, V–A gradient of 6 mmHg) that regularly followed blocked P waves (Fig. 3).

She was treated with a DDD pacemaker. No symptoms occurred during one year of follow up. Further TTE examinations showed no diastolic mitral regurgitation.

## Discussion

Competence of the mitral valve requires a temporally and spatially coordinated interaction of the mitral leaflets with the annulus, chordae tendinae and papillary muscles. Dysfunction of any of these components affects the normal systolic coaptation of the anterior and posterior leaflets and causes mitral regurgitation.

Mechanistically, mitral regurgitation (MR) is classified as either primary (intrinsic valve disease) or functional.^1^ Functional MR occurs in patients with a structurally normal valve (generally with restricted leaflet mobility), mitral annular dilation, and left ventricular remodelling.^2^ Functional MR is further classified as systolic and diastolic due to timing in the cardiac cycle.

Ischaemia is a well-known cause of functional MR.^3^ The underlying mechanism is apical tenting of structurally normal leaflets with subsequent papillary muscle displacement away from the mitral annulus plane.^4^ Eclipsed mitral regurgitation is an atypical form of sudden transient functional MR and is reported as sudden apical tenting of both leaflets in the absence of epicardial coronary artery stenosis and pre-existing LV systolic dysfunction or remodelling.^5^

Avierinos *et al*. reported severe mitral regurgitation with symptoms of heart failure that was induced by methylergonovine injection. Possible underlying mechanisms were epicardial focal spasm, diffuse epicardial vasoconstriction or microvascular dysfunction.

Rhythm and conduction disturbances are also common causes of mitral regurgitation. Electromechanical asynchrony can alter the left ventricular contraction pattern. Left bundle branch block (as in our first case) is an example of ventricular asynchrony related to functional mitral regurgitation.^6^,^7^

Functional mitral regurgitation in patients with left bundle block has multiple components, including asynchrony of the papillary muscles due to delay in ventricular conduction, which causes a delay in the contraction of the papillary muscles.^8^,^9^ The delayed movement of certain areas of the left ventricle (lateral wall or interventricular septum) leads to a reduction in the force of mitral valve closure due to the fall in systolic volume caused by the asynchronous mechanical contraction.

Long-term right ventricular (RV) apical pacing can also cause MR. It has been shown in canine models that RV pacing can increase mitral and tricuspid valve incompetence.^10^ While the incidence of MR appeared to be low at baseline, this study showed that MR can increase in the course of time due to permanent RV apical pacing. This holds true, especially in patients with pre-existing MR.

Left ventricular dyssynchrony can be considered as a cause of MR after long-term RV apical pacing. There are a few reported cases of acute severe MR as an immediate perioperative complication of pacemaker insertion, leading to acute haemodynamic deterioration. Rita *et al.* demonstrated that RVA pacing may immediately induce severe MR and acute cardiac failure, even in patients with preserved LV contraction.^11^ This case also shows RV outflow tract pacing improves MR compared with apical pacing, probably by improving ventricular dyssynchrony.

Diastolic mitral regurgitation (DMR) is a common phenomenon seen in patients with AV blocks, hypertrophic cardiomyopathy, advanced left ventricular systolic dysfunction, aortic valve disease, and in the presence of atrial fibrillation with long cardiac cycles. The haemodynamic mechanism leading to DMR is due to a positive ventricular-to-atrial (V–A) pressure gradient occurring during diastole.^12^,^13^ Because of the AV dyssynchrony, the AV pressure gradient reverses (ventricular pressure becomes higher than atrial), resulting in DMR in the presence of an incompletely closed mitral valve.^14^

## -Conclusion

These two cases illustrate different mechanisms of mitral regurgitation, which may have different haemodynamic consequences and clinical implications. The cases underline the importance of a high index of suspicion in patients with intermittent heart failure, and a careful analysis of echocardiographic images with simultaneous ECG, in order to delineate systolic and diastolic MR.
